# Psychosocial symptom networks and high-risk sexual behaviors among men who have sex with men: a network analysis

**DOI:** 10.1186/s40359-025-03550-x

**Published:** 2025-11-12

**Authors:** Nan Lin, Yuan Guo, Yun Chen, Yuting Yang, Haijiang Lin, Xiaoxiao Chen, Tingting Wang, Chaowei Fu, Shanling Wang, Jingyi Wang

**Affiliations:** 1 School of Public Health, NHC Key Laboratory of Health Technology Assessment, Fudan University, Shanghai, China; 2 Yale School of Nursing, Orange, CT 06477 USA; 3 Taizhou City Center for Disease Control and Prevention, Taizhou Zhejiang Province, China; 4 Taizhou Central Blood Station, Taizhou Zhejiang Province, China

**Keywords:** Psychosocial problems, High-risk sexual behaviors, Men who have sex with men, Network analysis

## Abstract

**Background:**

Psychosocial problems are prevalent among men who have sex with men (MSM) and are linked to high-risk sexual behaviors. Previous studies have often examined these factors separately, without considering their interconnections. Network analysis offers a way to explore these relationships, but has seldom been applied to MSM in the context of both mental health and sexual risk. This study aimed to: (1) model psychosocial symptom networks among MSM; (2) compare network structures across demographic subgroups; and (3) examine how central symptoms relate to high-risk sexual behaviors.

**Methods:**

A cross-sectional survey was conducted among 405 MSM in Taizhou, China, using venue-based and online snowball sampling. Psychosocial problems—including depression, anxiety, self-esteem, internalized homonegativity, loneliness, perceived social support, and sociosexual orientation—were assessed using validated self-report scales. High-risk sexual behavior was measured based on condom use and number of sexual partners. Network analysis was used to examine symptom-level associations, and network comparison tests explored differences across demographic subgroups. Associations between central symptoms and high-risk behaviors were examined using correlation and regression analyses.

**Results:**

Among 405 MSM, 12.4% reported moderate-to-severe depression and 10.1% moderate-to-severe anxiety. Strongest network connections appeared between depression and anxiety, and between loneliness and sociosexual desire. Central nodes included *‘feeling nervous’*, desire for uncommitted sexual relationships, *‘feeling tired’*, *‘lacking companionship’,* and *‘no good’*. Significant structural differences were found between participants < 33 years and those ≥ 33 years (*p* = 0.017), with older individuals showing generally weaker psychosocial connections. Central symptoms were significantly associated with risky sexual behaviors, especially in younger participants.

**Conclusions:**

Emotional distress, low self-esteem, loneliness, and sociosexual desire emerged as central psychosocial problems linked to high-risk sexual behaviors among MSM. Network structures differed by age, with younger MSM showing stronger interconnections. These findings highlight the need for age-specific, targeted interventions to reduce sexual risk and improve mental health among MSM.

**Supplementary Information:**

The online version contains supplementary material available at 10.1186/s40359-025-03550-x.

## Background

According to the Joint United Nations Programme on HIV/AIDS (UNAIDS) terminology guidelines, the term men who have sex with men (MSM) is used to describe cisgender or transgender men who engage in sexual activity with other men, regardless of whether they also have sex with women or identify as gay or bisexual [1]. Certain sexual practices, such as unprotected anal intercourse (UAI), multiple sexual partnerships (MSP), and engagement in commercial sex, are associated with heightened risk of Human Immunodeficiency Virus (HIV) transmission among MSM [2, 3]. Globally, MSM have been reported to face up to a 28-fold higher risk of HIV acquisition compared to heterosexual men [4]. It is well-established that engaging in high-risk sexual behaviors significantly increases the likelihood of acquiring and transmitting sexually transmitted infections (STIs), including HIV [5–7]. In the present study, we operationally defined our study population as MSM who reported anal intercourse, given its predominant role in HIV transmission risk. Other sexual behaviors, such as oral sex or mutual masturbation, were not included in the inclusion criteria.

MSM are often described as a marginalized and stigmatized population due to their sexual orientation and the elevated risk of HIV and other STIs associated with certain sexual practices [8–11]. According to the minority stress framework [12], stigma and social discrimination act as chronic stressors, contributing to psychosocial challenges among MSM. The prevalence of various psychosocial issues is significantly higher among MSM compared to their heterosexual counterparts [12–16]. These psychosocial problems typically manifest in two dimensions: mental health challenges and social or relational challenges. At the psychological level, MSM report higher rates of depression, anxiety, low self-esteem, and internalized homonegativity. At the social level, they are more likely to experience loneliness, limited social support, and challenges related to sociosexual orientation.

To date, extensive research has documented that depression and anxiety are prevalent psychological problems reported among MSM [17–22]. A meta-analysis showed that the prevalence of depression among MSM in China was 43.9% (95%CI: 36.9%−48.8%), and the rate was particularly high among MSM living with HIV [21]. Anxiety symptoms are also widespread, affecting approximately 32.2% of MSM according to another meta-analysis conducted in China [23]. Discrimination, minority stress, and concealment-related guilt have all been associated with the development of depression and anxiety among MSM [24–26]. Self-esteem, defined as an individual’s evaluation of personal worth, is another critical psychological factor frequently undermined by perceived and internalized stigma [17]. Existing studies in China report that 6%–7% of MSM experience low self-esteem [17, 27], reflecting challenges of self-acceptance linked to internalization of societal stigma [10]. Internalized homonegativity—characterized by negative self-perceptions associated with one’s sexual orientation—has also been found to be closely associated with depression, anxiety and low self-esteem among MSM [18, 28, 29]. These psychological problems are often intertwined, highlighting the complex nature of mental health within MSM populations.

Social relationship problems have long been linked to mental health challenges. Loneliness, a distressing emotion arising from unmet social relationship needs, is prevalent among MSM due to experiences of social exclusion and isolation. In China, MSM who experience loneliness are more likely to exhibit low self-esteem and moderate-to-severe depressive symptoms [17]. Furthermore, social support plays a crucial protective role, as lower levels of social support are strongly associated with higher levels of depression, anxiety, and loneliness [30, 31]. Sociosexual orientation is used to describe individual differences in the willingness to engage in casual sexual relationships. Some MSM exhibit an unrestricted sociosexual orientation, characterized by a greater openness to multiple or casual sexual partnerships [32]. Individuals with this orientation tend to engage in sexual activities without emotional attachment, favor one-night stands, and often have multiple sexual partners.

Psychosocial problems such as depression [33], anxiety [11], self-esteem [34], internalized homonegativity [35], loneliness [18], social support [36] and unrestricted sociosexual orientation [37] are closely linked to high-risk sexual behaviors. Psychological distress may drive MSM to engage in condomless sex or maintain multiple sexual partners as coping strategies [8–10]. Depression has been associated with a higher likelihood of condomless sex among MSM [18], though severe depressive symptoms may sometimes reduce sexual interest, reflecting a complex, nonlinear relationship [13]. Additionally, studies have indicated that low self-esteem is a significant factor contributing to UAI, a key determinant in the transmission of AIDS and other STIs [34, 38]. Social risk factors have also been shown to shape high-risk sexual behaviors by influencing patterns of partner seeking, sexual settings, relationship choices, and condom use decisions [30]. Social support is widely recognized as a protective factor, with men possessing stronger social support networks demonstrating a lower propensity for high-risk sexual behaviors [36]. In contrast, loneliness is linked to increased sexual risk-taking, particularly unprotected sex [18, 39].

While there has been substantial research documenting the prevalence of mental health problems among MSM, few studies have integrated these factors within a network framework. Previous studies have often examined the relationships between mental health factors and sexual behaviors in isolation [40], without considering their complex interactions. Recent research suggests that the psychosocial issues may be better understood as interconnected symptoms within a network [41]. Network analysis has gained increasing recognition as a powerful method for capturing the complexity of mental health, revealing how symptoms such as depression, anxiety, and self-esteem are interrelated rather than independent. This approach enables more targeted interventions by identifying central symptoms—those most strongly connected to others in the network [42]. Given the known comorbidity and syndemic relationship between psychosocial issues [11, 15, 34], network analysis provides a valuable tool for mapping these dynamics, offering both visual and quantitative insights [43]. Despite its growing use in applied psychology, network analysis has rarely been applied to MSM populations to simultaneously examine mental health and sexual risk behaviors. Moreover, some salient factors for MSM, such as internalized homonegativity and sociosexual orientation, have received limited attention within psychosocial network research.

In addition, demographic groups such as age, marital status, education level, and sexual orientation may exhibit distinct psychosocial characteristics and patterns of high-risk sexual behaviors. Prior studies have shown age-related differences in sexual risk behaviors. Compared with younger individuals, middle-aged and older adults were more likely to report having multiple partners, using condoms inconsistently, and reducing condom use after HIV diagnosis [44]. However, the extent to which demographic factors, particularly age, shape the structure and dynamics of psychosocial networks and differentially influence their associations with high-risk sexual behaviors remains insufficiently understood among MSM. Network analysis, with its focus on systemic and multidimensional relationships, offers a promising approach to explore these complex interactions and to inform the design of more personalized interventions.

To address the identified research gap and better understand the complex interplay between psychosocial problems and high-risk sexual behaviors among MSM, a cross-sectional survey was conducted in Taizhou, China, with three primary objectives: (1) to construct a comprehensive network model of psychosocial problems, including depression, anxiety, self-esteem, internalized homonegativity, loneliness, social support, and sociosexual orientation; (2) to compare psychosocial network structures across MSM subgroups defined by age, marital status, educational level, and sexual orientation, identifying subgroup-specific patterns; and (3) to examine the associations between central psychosocial symptoms within these networks and high-risk sexual behaviors across different demographic subgroups. Together, these analyses aim to identify critical psychosocial factors and interrelationships, providing an evidence base for developing targeted interventions to improve mental health and reduce sexual risk behaviors among MSM.

## Methods

### Participants

A cross-sectional survey was conducted among MSM from January to August 2022 in three districts (Huangyan, Jiaojiang, and Luqiao) of Taizhou, Zhejiang Province, China. Participants were eligible if they: (1) were biologically male; (2) aged 15 years or older; (3) had previously engaged in anal sex with another man; (4) had no severe cognitive or communication impairments; and (5) provided informed consent to participate. Participants were identified as MSM based on self-reported sexual behavior. Specifically, eligibility required reporting a history of anal sex with another man, consistent with the behavior-based definition of MSM recommended by UNAIDS [1]. This ensured that all participants met the behavioral criteria for inclusion, rather than being classified solely on the basis of sexual identity. The study was approved by the Ethics Committee of Taizhou Central Hospital (2022L-01–18), and all methods were carried out in accordance with relevant guidelines and regulations.

Due to the hidden nature of the MSM population, participants were recruited using a combination of convenience and snowball sampling methods. Initially, recruitment was carried out at venues frequently visited by MSM, including bars, nightclubs, saunas, and public parks. To ensure privacy and confidentiality, participants completed questionnaires individually in undisturbed areas within these venues. Trained research staff provided clarification and assistance upon request. Additionally, online advertisements were posted on popular MSM-oriented platforms within Zhejiang Province, including forums, chat rooms, and social networking applications. Participants recruited through these platforms were encouraged to share the questionnaire with their MSM peers, continuing until the desired sample size was reached. However, as both venue-based and online snowball sampling may disproportionately recruit more socially connected MSM, this approach could introduce selection bias, which should be considered when interpreting the findings.

The target sample size was determined using the Monte Carlo method for network model estimation, implemented through the “powerly” R package [45]. This approach employs recursive simulation, curve fitting, and stratified bootstrapping to estimate the sample size required for adequate statistical power, accounting for network complexity. Based on these simulations, our calculations confirmed that the sample size was adequate to achieve sufficient statistical power for robust estimation. In total, 534 MSM completed the psychosocial questionnaire with informed consent. For the network analysis, listwise deletion was applied: participants with missing data on any of the key variables were excluded. The key variables included seven psychosocial constructs (depression, anxiety, self-esteem, internalized homonegativity, loneliness, perceived social support, and sociosexual orientation) as well as high-risk sexual behaviors. As the proportion of missing data for most variables was below 10%, the impact of listwise deletion on the final sample was limited. After applying this criterion, the final analytical sample consisted of 405 participants.

### Measures

#### Sociodemographic characteristics

The sociodemographic variables included age, marital status (never married or previously married, married or cohabiting), educational level (middle school or below, high school or technical school, college or above), and sexual orientation (homosexual, bisexual/heterosexual/unsure).

#### Psychosocial problems

##### Depressive symptoms

Depressive symptoms among the MSM population were measured using the Patient Health Questionnaire-9 (PHQ-9) scale, a widely utilized and effective self-report instrument designed to assess depression over the preceding two weeks [46]. The PHQ-9 consists of nine items, each corresponding to one of the nine DSM-IV diagnostic criteria for depression. Respondents rate their experiences on a four-point scale: "not at all" (0 points), "several days" (1 point), "more than half the days" (2 points), and "nearly every day" (3 points). The possible total score ranges from 0 to 27, with higher scores indicating more severe depressive symptoms. The Chinese version of PHQ-9 has been demonstrated as a valid and reliable tool to screen depression [47], and the Cronbach’s alpha was 0.88 in this study.

##### Anxiety symptoms

Anxiety levels were assessed using the Generalized Anxiety Disorder 7-item scale (GAD-7) [48]. This scale consists of seven items, each rated on a four-point scale: "not at all" (0 points), "several days" (1 point), "more than half the days" (2 points), and "nearly every day" (3 points). The total score is the sum of the seven items, with a higher score indicating greater anxiety. Prior research has established the reliability and validity of the Chinese version of the GAD-7 [49]. In this study, the scale also demonstrated excellent internal consistency (Cronbach’s alpha 0.91).

##### Self-esteem

Self-esteem was assessed using the Rosenberg Self-Esteem Scale (RSES), one of the most widely used instruments for measuring self-evaluation of personal worth [50]. The RSES consists of 10 items, five of which are positively worded and five reverse-scored. Respondents rate each item on a four-point scale: "strongly agree" (3 points), "agree" (2 points), "disagree" (1 point), and "strongly disagree" (0 points). The total possible score ranges from 0 to 30, with higher scores indicating higher self-esteem. Previous research validating the Chinese version of the RSES has demonstrated excellent reliability, with Cronbach’s α ranging from 0.911 to 0.942, and omega (ω) values ranging from 0.915 to 0.944 [51, 52]. The Cronbach’s alpha was 0.85 in this study.

##### Internalized homonegativity

The Short Internalized Homonegativity Scale (SIHS) is a commonly used instrument for measuring internalized homonegativity—namely, the internalization of negative attitudes and assumptions about homosexuality by gay individuals themselves [52]. The SIHS consists of eight items, which are divided into three subscales: "Social Comfort with Gay Men," "Public Identification as Gay," and "Personal Comfort with a Gay Identity". Higher scores on the scale indicate greater acceptance of one’s sexual orientation and lower levels of internalized homonegativity. Internalized homonegativity was assessed using the Chinese version of the SIHS, which has been culturally adapted and validated among Chinese gay men [53, 54]. The Cronbach’s alpha was 0.76 in this study.

##### Loneliness

The 3-item UCLA Loneliness Scale (UCLA-3) was used to assess feelings of loneliness [55]. This scale consists of three items, each rated on a three-point scale: "hardly ever" (1 point), "some of the time" (2 points), and "often" (3 points). Scores of the scale range from 3 to 9, with higher scores indicating higher levels of perceived loneliness. The Chinese version of the UCLA-3 has been shown to effectively track variations in loneliness across time and is widely used in Chinese research [56]. In the present study, the scale demonstrated good internal consistency (Cronbach’s alpha 0.84).

##### Perceived social support

The Multidimensional Scale of Perceived Social Support (MSPSS) was used to assess individuals' perceived support [50]. The MSPSS consists of 12 items designed to evaluate perceived social support from three sources: Family, Friends, and Significant Others. Each item is rated on a 7-point Likert scale, ranging from "very strongly disagree" (1 point) to "very strongly agree" (7 points). The average score for each subscale was calculated separately, with higher scores indicating greater levels of social support. Previous research validating the Chinese version of the MSPSS has demonstrated excellent reliability, with an internal consistency coefficient of 0.91 and test–retest reliability ranging from 0.84 to 0.91 [57]. The Cronbach’s alpha was 0.93 in this study.

##### Sociosexual orientation

The revised Sociosexual Orientation Inventory (SOI-R) was used to assess people’s willingness to engage in uncommitted sexual relationships [37]. The scale comprises 9 items, which are categorized into three subscales: "Behavior," "Attitudes," and "Desire." Only the latter two dimensions were included in the psychosocial network analysis. This decision was made for both theoretical and methodological reasons: the Behavior dimension primarily captures past sexual activity (e.g., number of sexual partners), which overlaps conceptually and empirically with our outcome measure of high-risk sexual behaviors. Including it in the network could therefore inflate associations due to construct redundancy. In contrast, the Attitude and Desire dimensions represent underlying psychological dispositions toward uncommitted sex, making them more appropriate for mapping psychosocial interrelations in the network.

The Attitude dimension consists of three items that assess one’s disposition toward uncommitted sexual behavior, reflecting the desired level of emotional closeness before engaging in sex and the moral evaluation of such behavior. The Desire dimension also comprises three items and measures interest in uncommitted sexual activity, characterized by heightened sexual desire often accompanied by subjective arousal and sexual fantasies. Unlike general sexual desire, unrestricted sociosexual desire refers to sexual attraction to potential partners outside of committed relationships. We employed the 5-point response format, which is considered more suitable for participants with diverse educational backgrounds and survey familiarity. For Attitude items, responses ranged from 1 (strongly disagree) to 5 (strongly agree). For Desire items, responses ranged from 1 (never) to 5 (nearly every day). After reverse-coding item 6, scores were averaged within each subscale, with higher scores indicating a greater degree of unrestricted sociosexual orientation. Sociosexual orientation was assessed using the Chinese version of the SOI-R, which is officially available from the scale developers and has demonstrated good reliability and validity in Chinese samples [58, 59]. The Cronbach’s alpha was 0.84 in this study.

#### High-risk sexual behaviors

High-risk sexual behavior was assessed using two items: (1) the frequency of condom use during anal sex with male partners in the past six months, with response options coded as: 1 = no sexual activity in the past six months, 2 = always used condoms, 3 = sometimes used condoms, and 4 = never used condoms; and (2) the number of sexual partners in the past 12 months, as measured by an item from the Behavior dimension of the SOI-R. For this item, the response options were 0, 1, 2–3, 4–7, and 8 or more, coded from 1 to 5, with higher scores indicating a greater number of partners. A composite score was calculated by combining the two items, with higher scores reflecting a greater likelihood of engaging in high-risk sexual behaviors.

### Statistical analysis

#### Descriptive analysis

Descriptive analyses were conducted for participants’ sociodemographic characteristics, psychosocial problems, and high-risk sexual behaviors. Continuous variables were summarized using means and standard deviations, while categorical variables were presented as frequencies and percentages. Independent samples t-tests and chi-square tests (χ2) were used to compare the characteristics of MSM aged < 33 years and those aged ≥ 33 years. The cut-off of 33 years was chosen because it approximated the mean age of the sample (M = 33.4, SD = 11.5), allowing for a balanced division of participants into younger and older groups for comparison.

#### Network estimation and centrality

Network analysis is an emerging methodology in applied psychology that conceptualizes psychological constructs as systems of interacting components, rather than as manifestations of latent variables. By modeling these components and their interconnections, network analysis allows for the identification of central psychosocial factors that may play a critical role in the maintenance of mental health and behavioral outcomes. Network analysis employs mathematical graphs to model the interconnections between symptoms, where each symptom is represented as a node and the relationships between them as edges. The thickness of each edge reflects the strength of the association. Psychosocial problems included in the network structure analysis were depression, anxiety, self-esteem, internalized homonegativity, loneliness, perceived social support, and sociosexual orientation. All individual items from the scales measuring depressive symptoms, anxiety symptoms, loneliness, and self-esteem were treated as nodes in the network. In addition, total scores from each dimension of perceived social support (three dimensions), sociosexual orientation (two dimensions), and internalized homonegativity (three dimensions) were also included as network nodes. The network was estimated using the "bootnet" package in R and visualized with the "qgraph" package [60]. Pairwise Spearman correlations and sparse Gaussian graphical models with graphical lasso were used to estimate the associations between psychosocial problems among MSM. Spearman correlation was chosen over Pearson because most psychosocial variables in this study were measured using Likert-type scales, which are ordinal rather than truly continuous, and the relationships between them may not be strictly linear. As a rank-based, non-parametric method, Spearman correlation is more robust to these conditions and therefore more appropriate for capturing the associations among psychosocial problems and high-risk sexual behaviors.

Centrality indices are commonly used to assess the structural importance of nodes within a network. Symptoms with higher centrality are those most strongly connected to others, indicating their critical role in the overall network [61]. To assess node centrality within the psychosocial network, strength, betweenness, closeness, and expected influence were calculated and visualized [62, 63]. Strength was defined as the total sum of the absolute edge weights directly connected to a specific node, with higher-strength nodes potentially triggering the activation of other symptoms. Betweenness measured the extent to which a node lay along the shortest paths between pairs of other nodes, suggesting that nodes with high betweenness could act as bridges linking different symptom clusters within the network. Closeness referred to the inverse of the average shortest path length from a given node to all other nodes, meaning that a node with high closeness might be more directly connected to a broader range of symptoms. Expected Influence (EI), a more recent centrality metric, expanded on strength by accounting for both positive and negative edge weights when evaluating a node’s influence in the network [64]. Network stability was evaluated using a case-dropping subset bootstrap approach with 1,000 iterations. The correlation stability coefficient was used to quantify the robustness of the centrality estimates, with values above 0.5 generally considered indicative of a stable network.

#### Network comparison

To compare the psychosocial network structures of MSM across different age groups, marital statuses, educational levels, and sexual orientations, the “NetworkComparisonTest” package in R was employed [65]. A permutation test with 1,000 iterations was conducted to examine both global and local differences in network edges. Global network structure invariance was assessed by evaluating the maximum difference in edge weights between two networks. Global strength differences were quantified by comparing the weighted sum of all absolute edge values across networks. Local differences were assessed by testing the invariance of each individual edge between the two networks.

#### Association between central symptoms and high-risk sexual behavior

To examine whether central symptoms in the psychosocial network were more strongly associated with high-risk sexual behavior, Spearman rank correlation analyses were conducted between each network node and the high-risk behavior score. Higher correlation coefficients indicated a stronger association between the symptom and high-risk sexual behavior. The relationship between centrality and correlation coefficients was evaluated using locally weighted scatterplot smoothing (LOWESS). Linear regression analysis was performed to assess whether nodes with higher centrality values were more strongly associated with high-risk sexual behavior.

## Results

The characteristics of the participants (*n* = 405) are illustrated in Table 1. Of the participants with a mean age of 33.4 years (range 16–76), 66.4% were never married or previously married, 33.1% had middle school or lower education, and 58.3% were self-identified as homosexual. Furthermore, 50 (12.4%) participants exceeded the cut-off score of 10 for moderate-to-severe depression and 41 (10.1%) exceeded the cut-off score of 10 for moderate-to-severe anxiety. In total, 27 (6.7%) participants had both clinically relevant depressive and anxiety symptoms. A high percentage of MSM suffered from loneliness (25.7%), 19.3% had unprotected anal intercourse in the last six months and 68.9% had multiple sexual partners in the past 12 months. Compared with younger participants, a higher proportion of MSM aged 33 and older were married or cohabiting, had lower education, and were self-identified as bisexual. The older participants also reported lower self-esteem, poorer social support from family, friends, and significant others, as well as a more positive attitude towards unrestricted sociosexual orientation, but a lower desire for uncommitted sexual relationships.

**Table 1:** Participant characteristics

	Total sample (*n* = 405)	Age < 33 old (*n* = 213)	Age ≥ 33 old (*n* = 192)	*P* value
Sociodemographic variables				
Age (16–76)	33.4 (11.5)	24.3 (4.3)	43.6 (7.9)	< 0.001
Marital status, n (%)				< 0.001
Never married or previously married	269 (66.4)	199 (93.4)	70 (36.5)	
Married or cohabiting	136 (33.6)	14 (6.6)	122 (63.5)	
Educational level, n (%)				< 0.001
Middle school or below	134 (33.1)	25 (11.7)	109 (56.8)	
High school or technical school	155 (38.3)	97 (45.5)	58 (30.2)	
College or above	116 (28.6)	91 (42.7)	25 (13.0)	
Sexual orientation, n (%)				< 0.001
Homosexual	236 (58.3)	167 (78.4)	69 (35.9)	
Bisexual/heterosexual/unsure	169 (41.7)	46 (21.6)	123 (64.1)	
Psychosocial problems				
Depression (0–27)	4.6 (4.4)	4.7 (4.5)	4.5 (4.2)	0.547
Anxiety (0–21)	4.1 (4.1)	4.2 (4.0)	4.0 (4.2)	0.720
Loneliness (3–9)	4.3 (1.5)	4.2 (1.6)	4.3 (1.4)	0.671
Self-esteem (0–30)	20.3 (4.2)	20.8 (4.7)	19.8 (3.6)	0.014
Perceived social support				
Family support (1–7)	4.7 (1.0)	4.8 (0.9)	4.5 (1.0)	0.003
Friends support (1–7)	4.7 (1.0)	4.8 (0.9)	4.5 (1.0)	< 0.001
Significant others support (1–7)	4.7 (1.0)	4.8 (0.9)	4.5 (1.0)	< 0.001
Sociosexual orientation				
Attitude (3–15)	8.2 (2.1)	7.9 (2.2)	8.7 (2.0)	< 0.001
Desire (3–15)	7.3 (3.1)	7.7 (3.2)	7.0 (2.9)	0.021
Internalized homophobia				
Social comfort with Gay Men (3–21)	12.6 (2.5)	12.4 (2.5)	12.8 (2.5)	0.107
Public Identification as Gay (2–14)	8.0 (2.4)	7.9 (2.4)	8.2 (2.4)	0.307
Personal Comfort with a Gay Identity (3–21)	13.4 (2.8)	13.3 (2.9)	13.6 (2.7)	0.333
High-risk sexual behaviors				
Unprotected anal intercourse (1–4)	1.9 (0.8)	1.9 (0.7)	1.9 (0.8)	0.628
Multiple Sexual Partners (1–5)	3.0 (1.2)	2.9 (1.1)	3.1 (1.2)	0.144

^*^Continuous variables were presented as mean (standard deviation), and categorical variables were presented as number (percentage)

### Network estimation

**Fig. 1:**
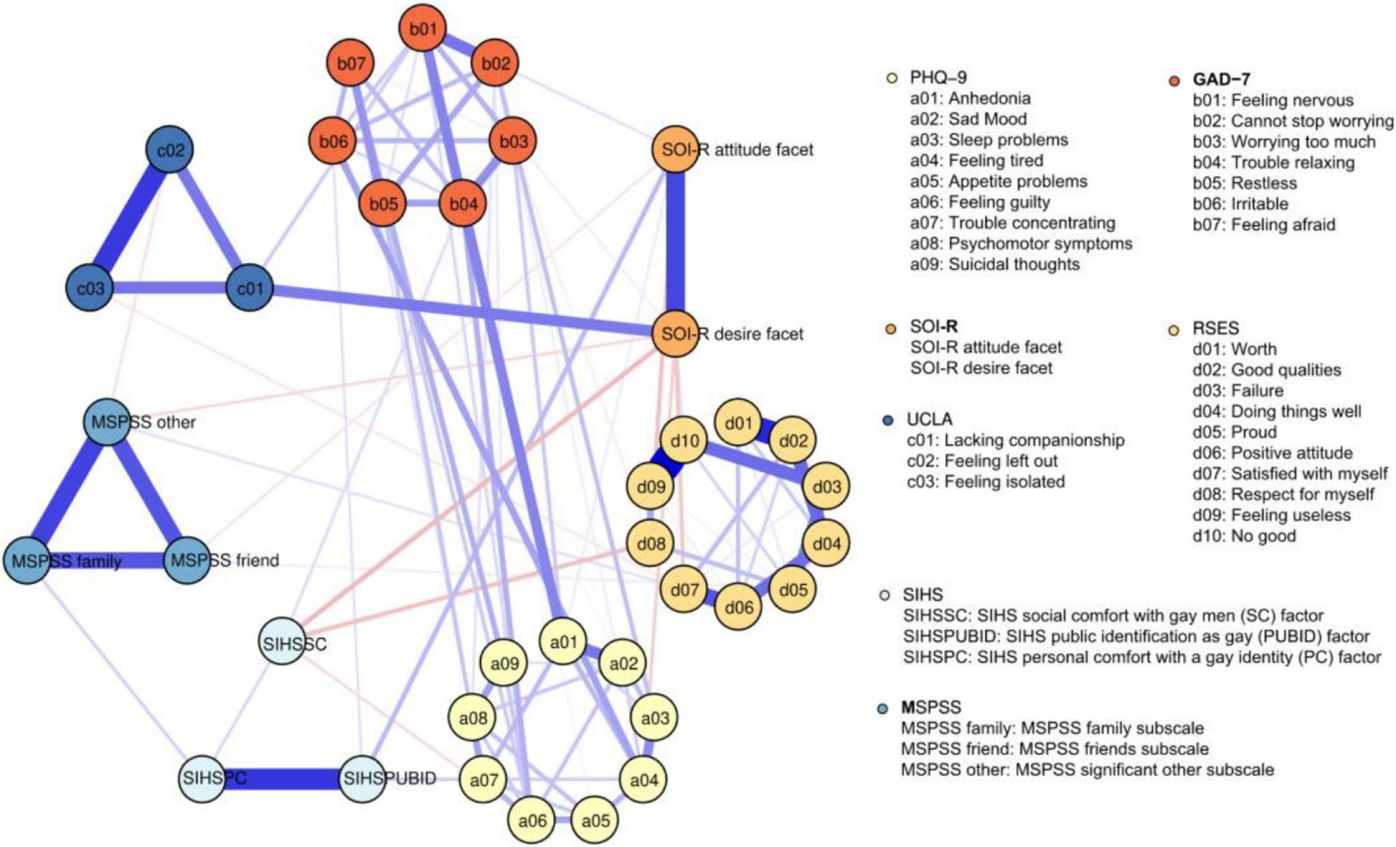
Psychosocial network structure in the MSM. The light yellow nodes denote depressive symptoms (PHQ-9 items), the red nodes denote anxiety symptoms (GAD-7 items), the blue nodes denote loneliness (UCLA-3 items), the yellow nodes denote self-esteem (RSES items), the light blue nodes denote perceived social support (MSPSS subscales), the orange nodes denote unrestricted sociosexual orientation (SOI-R subscales), and the grey nodes denote internalized homonegativity (SIHS subscales). The blue edges denote the positive correlations and the red edges denote the negative correlations

We estimated the overall network including separate items for depressive symptoms, anxiety symptoms, loneliness, self-esteem, and subscale scores for perceived social support from family, friends and significant other, attitude and desire scores for unrestricted sociosexual orientation, and three subscale scores for internalized homonegativity (Fig. 1). The items of these measures and their reference names were listed in Table S1. Within the overall network, we observed strong connections between the items of depressive symptoms and anxiety symptoms, particularly between *‘anhedonia’* and *‘feeling nervous’* (edge weight = 0.23). The loneliness item *‘lacking companionship’* was positively connected with the desire for uncommitted sexual relationships (edge weight = 0.25) and the anxiety item *‘feeling nervous’* (edge weight = 0.09). Among the items of self-esteem, *‘satisfied with myself’* was negatively linked to the desire for uncommitted sexual relationships (edge weight = −0.1), and *‘respect for myself’* was negatively associated with the attitude towards uncommitted sexual relationships (edge weight = −0.1) and social comfort with gay men (SIHSSC) (edge weight = −0.11). Furthermore, social comfort with gay men (SIHSSC) was negatively connected with the desire for uncommitted sexual relationships (edge weight = −0.12), while public identification as gay (SIHSPUBID) was positively associated with the attitude towards uncommitted sexual relationships (edge weight = −0.11), and personal comfort with a gay identity (SIHSPC) was positively linked to family social support (edge weight = 0.08). Detailed edge weights for all the edges in the network can be found in Table S2. Items of depressive symptoms were on average explained for 52.3% by variables in the network that were directly connected to them, for items of anxiety symptoms 61.0%, for loneliness items 54.8% and for self-esteem items 56.5%. Explained variance for perceived social support subscale scores was on average 63.6%, for unrestricted sociosexual orientation 39.9%, and for internalized homonegativity 38.6%.

Stability of the network was evaluated using the bootstrap method. The edge weights in the current sample were largely consistent with the bootstrapped sample, indicating relatively stable estimates (see Figure S1). The correlation stability coefficients exceeded 0.5 even using 30% of the cases, indicating that the network structure was stable.

### Network comparison

**Fig. 2:**
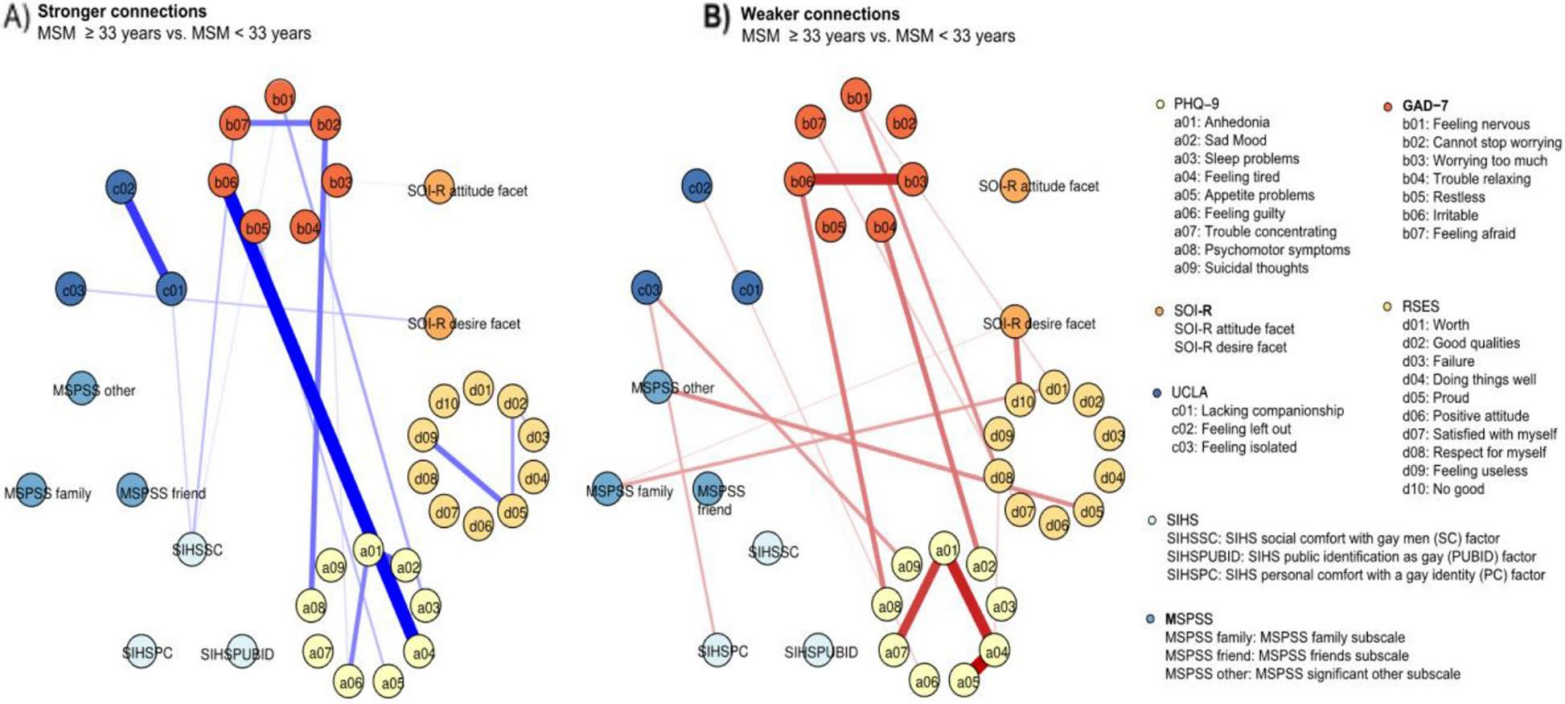
Edges exhibiting significant differences between participants ≥ 33 years and those < 33 years (participants < 33 years as reference). The light yellow nodes denote depressive symptoms (PHQ-9 items), the red nodes denote anxiety symptoms (GAD-7 items), the blue nodes denote loneliness (UCLA-3 items), the yellow nodes denote self-esteem (RSES items), the light blue nodes denote perceived social support (MSPSS subscales), the orange nodes denote unrestricted sociosexual orientation (SOI-R subscales), and the grey nodes denote internalized homonegativity (SIHS subscales). The blue edges denote stronger correlations between items and subscales in the older MSM network when compared with those in the younger MSM network, and the red edges denote weaker correlations. **A** Stronger correlations in participants ≥ 33 years compared with those < 33 years; **B** Weaker correlations in participants ≥ 33 years compared with those < 33 years

We conducted network comparison tests across four sociodemographic variables: age, marital status, educational level, and sexual orientation. Among these, only age groups showed a significant global difference in network structure, with participants aged < 33 years differing from those ≥ 33 years (*p* = 0.017). No significant global differences were found for marital status, educational level, or sexual orientation. In addition, several local edge differences were observed between the two age groups, and the statistically significant higher and lower correlations are visualized separately in Fig. 2 (*p* < 0.05).

When comparing participants ≥ 33 years with those < 33 years, we observed differences in connections between items of depressive symptoms and items of anxiety symptoms. In MSM ≥ 33 years, there were stronger connections between *‘feeling tired’* and *‘irritable’*, between *‘psychomotor symptoms’* and *‘cannot stop worrying’* and between *‘sleep problems’* and *‘feeling nervous’*, whereas the connections between *‘psychomotor symptoms’* and *‘irritable’* and between *‘sad mood’* and *‘trouble relaxing’* were weaker. The connections between items of emotional symptoms and the other psychosocial problems generally decreased in the older MSM, such as the relations between *‘suicidal thoughts’* and *‘feeling isolated’* and between *‘feeling nervous’* and *‘respect for myself’*. Furthermore, we observed that a number of associations between loneliness, self-esteem, social support, unrestricted sociosexual orientation and internalized homonegativity were significantly weaker in older MSM when compared to the younger group. For example, there were weaker connections between the following nodes: social support from family – *‘worth’*, social support from significant other – ‘proud’, *‘no good’* – desire for uncommitted sexual relationships, and *‘feeling isolated’* – personal comfort with a gay identity.

### Network centrality

**Fig. 3:**
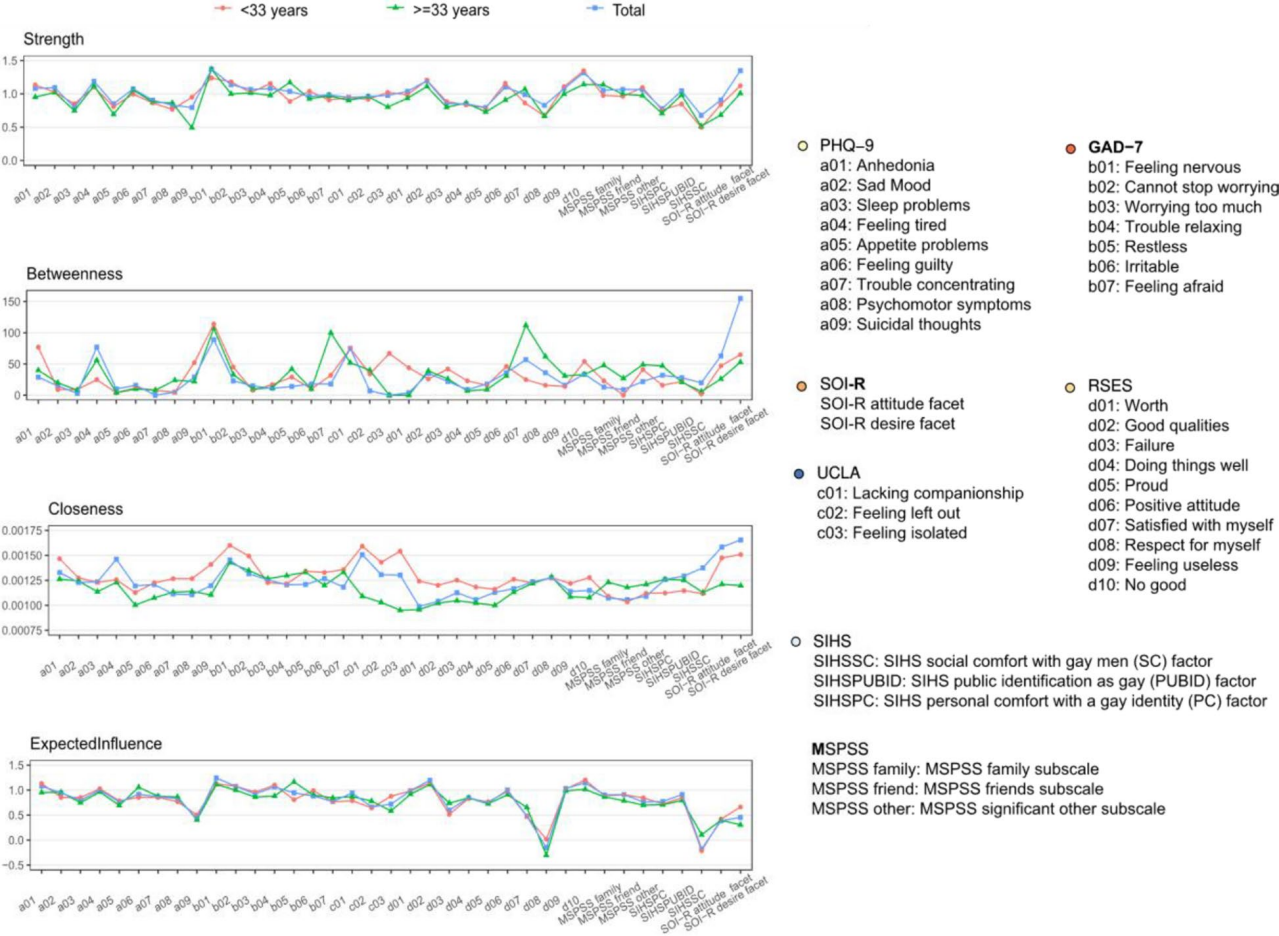
Network centrality of each node in the networks among the total sample, participants < 33 years and those ≥ 33 years. Strength is the sum of the absolute edge weights directly linked to a focal node in the network. Betweenness refers to the degree that a focal node lies on the shortest path between another two nodes. Closeness is defined as the inverse of the average shortest path length from a focal node to other nodes. Expected influence is an index of strength that accurately calculates a node’s linkage including positive and negative edges

Figure 3 shows the network centrality of each item and subscale in the total sample, participants < 33 years and those ≥ 33 years. In the total sample, *‘feeling nervous’*, desire for uncommitted sexual relationships, *‘feeling tired’*, *‘lacking companionship’* and *‘no good’* exhibited high network centrality. These nodes were strongly connected with other psychosocial problems in the network, suggesting that they represent core aspects of psychological distress and social functioning among MSM. For instance, *‘no good’* (at times I think that I am no good at all) reflects negative self-evaluation within self-esteem, a construct closely linked to mental health vulnerability. *‘Feeling tired’* (feeling tired or having little energy) indicates somatic complaints often associated with emotional distress, while *‘lacking companionship’* highlights the centrality of loneliness and unmet social needs.

In subgroup analyses, *‘suicidal thoughts’*, loneliness items and desire for uncommitted sexual relationships showed higher centrality values in participants < 33 years compared to those ≥ 33 years, suggesting that internalizing symptoms and sociosexual orientation are particularly salient in younger MSM. In contrast, *‘feeling afraid’* and *‘satisfied with myself’* showed greater betweenness in participants ≥ 33 years, indicating their potential role as bridging nodes that connect different psychosocial items in older MSM.

### Connection value of central symptoms

Spearman’s correlations were computed between each psychosocial problem and risky sexual behaviors (see Table 2). Behavior items were coded such that higher scores indicate a higher level of risky sexual behaviors. A high absolute correlation indicates that the severity of the problem was associated with risky sexual behaviors. Depressive symptoms, anxiety symptoms, self-esteem, perceived social support and acceptance of homosexuality negatively correlated with risky sexual behaviors, indicating that individuals with more severe emotional symptoms, higher self-esteem, more social support and greater acceptance of homosexuality were less likely to have risky sexual behaviors. By comparison, the correlation values were positive for loneliness and unrestricted sociosexual orientation, indicating that individuals with more severe loneliness and higher levels of attitude and desire for uncommitted sexual relationships were more likely to have risky sexual behaviors. We refer to the values in Table 2 as *connection values*. Higher absolute values indicate that the problem has a strong connection with risky sexual behaviors.

**Table 2:** Connection value of each node

Nodes	Total sample	Age < 33 years	Age ≥ 33 years
Anhedonia	−0.25	−0.31	−0.20
Sad Mood	−0.23	−0.31	−0.14
Sleep problems	−0.29	−0.31	−0.28
Feeling tired	−0.32	−0.31	−0.33
Appetite problems	−0.16	−0.26	−0.05
Feeling guilty	−0.20	−0.27	−0.14
Trouble concentrating	−0.18	−0.28	−0.08
Psychomotor symptoms	−0.13	−0.21	−0.06
Suicidal thoughts	−0.14	−0.11	−0.18
Feeling nervous	−0.24	−0.28	−0.19
Cannot stop worrying	−0.24	−0.29	−0.19
Worrying too much	−0.24	−0.23	−0.26
Trouble relaxing	−0.29	−0.28	−0.29
Restless	−0.21	−0.23	−0.20
Irritable	−0.26	−0.19	−0.33
Feeling afraid	−0.22	−0.24	−0.20
Lacking companionship	0.04	−0.03	0.12
Feeling left out	0.05	−0.14	0.24
Feeling isolated	0.07	−0.12	0.27
Worth	−0.08	−0.04	−0.13
Good qualities	−0.12	−0.08	−0.15
Failure	−0.09	0.01	−0.19
Doing things well	−0.07	0.00	−0.16
Proud	−0.09	−0.01	−0.19
Positive attitude	−0.07	−0.02	−0.12
Satisfied with myself	−0.11	−0.02	−0.21
Respect for myself	0.11	0.01	0.22
Feeling useless	−0.03	0.00	−0.07
No good	−0.05	−0.04	−0.06
MSPSS family subscale	−0.27	−0.18	−0.34
MSPSS friends subscale	−0.34	−0.22	−0.45
MSPSS significant other subscale	−0.32	−0.20	−0.41
SOI-R attitude facet	0.12	0.03	0.22
SOI-R desire facet	0.34	0.23	0.49
SIHS social comfort with gay men (SC) factor	−0.07	0.04	−0.19
SIHS public identification as gay (PUBID) factor	−0.24	−0.24	−0.24
SIHS personal comfort with a gay identity (PC) factor	−0.20	−0.24	−0.15

Connection value was determined by computing Spearman’s correlation between each node and risky sexual behaviors

**Fig. 4:**
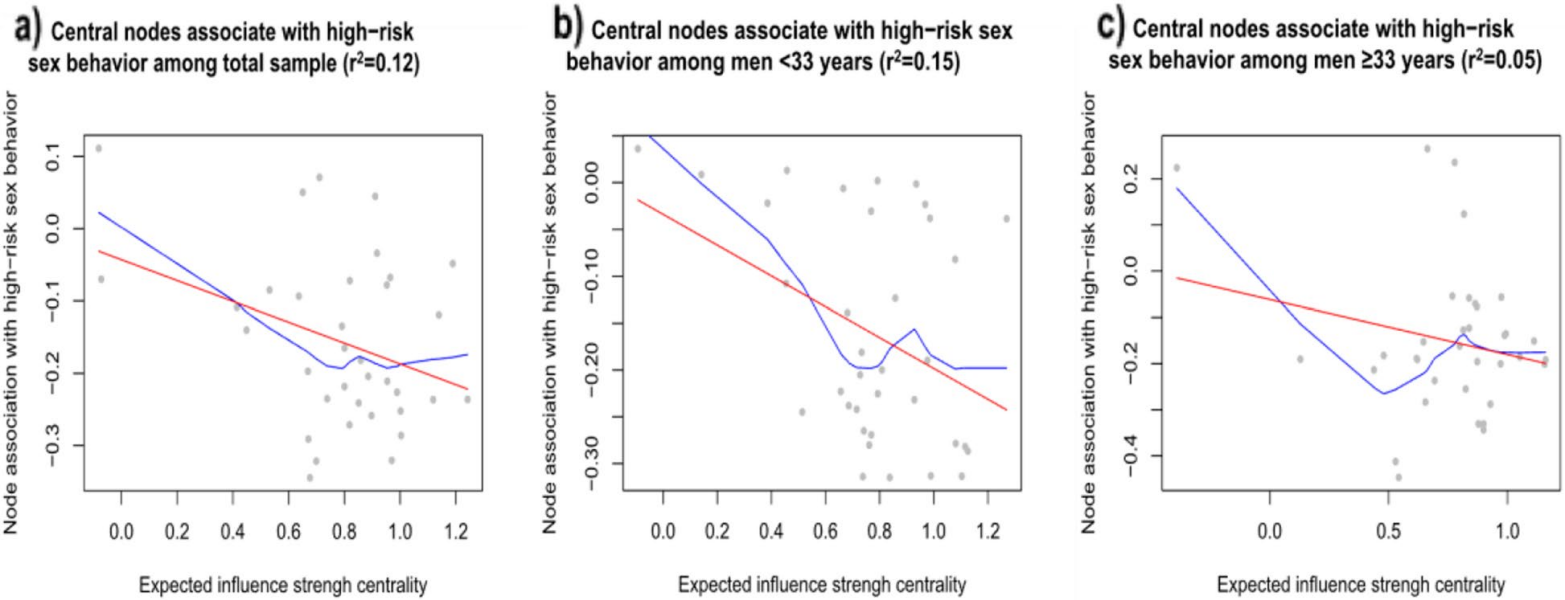
Association between centrality of psychosocial problems and connection values for risky sexual behaviors. Each point represents a psychosocial problem (i.e., node) in the network. A point towards the right on the x-axis indicates a problem which was highly central. A point large on the y-axis represents a problem which has high connection value. The results verified an association between a problem’s centrality and the connection value of that problem with risky sexual behaviors. **a**) Central nodes associated with high-risk sexual behavior in the total sample (r² = 0.12); **b**) Central nodes associated with high-risk sexual behavior among men aged <33 years (r² = 0.15); **c**) Central nodes associated with high-risk sexual behavior among men aged ≥33 years (r² = 0.05)

We then examined whether the centrality of psychosocial problems was associated with connection values. Linear regression results revealed that centrality of problems was significantly associated with connection values for risky sexual behaviors among the total sample (β = −0.144, *p* = 0.039) and participants < 33 years (β = −0.164, *p* = 0.022), but not among those ≥ 33 years (β = −0.119, *p* = 0.218). The results of this analysis are presented in Fig. 4. The estimation results of LOWESS in Fig. 4 also show that the general tendency between the expected influence strength centrality and the connection value was negative. To properly understand this figure, it is helpful to point out that each point represents a psychosocial problem (i.e., node) in the network. A point towards the right on the x-axis indicates a problem which was highly central. A point large on the y-axis represents a problem which has high connection value. The results thus verified an association between a problem’s centrality and the connection value of that problem with risky sexual behaviors, and the association was stronger in younger participants compared with the older ones.

## Discussion

This study employed network analysis to explore the interrelations among depression, anxiety, self-esteem, internalized homonegativity, loneliness, perceived social support, and sociosexual orientation within the MSM population in Taizhou, China. The study also identified central symptoms within the psychosocial network structure and examined their associations with high-risk sexual behaviors. The results revealed significant associations among these psychosocial problems. *‘Feeling nervous’*, desire for uncommitted sexual relationships, *‘feeling tired’*, *‘lacking companionship’*, and *‘no good’* emerged as central symptoms in the network and were significantly associated with high-risk sexual behaviors. Subgroup analyses by age indicated significant differences in the psychosocial network structures across different age groups of MSM.

In this study, 12.4% and 10.1% of MSM reported moderate or higher levels of depressive and anxiety symptoms, respectively—rates lower than those reported in prior studies. For example, depression prevalence among MSM was 27.4% in a UK study [22], 24.9% in a Brazilian multicity study [25], and 36% among newly diagnosed HIV-positive MSM in China [66]. Variations in prevalence may be attributed to differences in sample demographics, study periods, measurement tools, and HIV infection status. Regarding sexual risk behaviors, 19.3% of participants reported engaging in condomless anal intercourse in the past six months, and 68.9% reported having multiple sexual partners in the past 12 months. These findings are consistent with international data. A systematic review of MSM in high-income countries documented a substantial increase in condomless anal sex, rising from approximately 35% in 1990 to 55% by 2012, alongside a shift in sexual partner trends: little change between 1992 and 2002, with about 40% of MSM reporting multiple partners, followed by a substantial increase to over 60% during 2003–2013 [67]. These findings highlight ongoing psychological burdens and sexual risk behaviors among MSM. However, such historical patterns should not be attributed solely to behavioral changes, as advances in HIV/STI testing, treatment, and prevention technologies during this period also contributed to changing perceptions of risk and condom use practices. Further research is warranted to elucidate the psychological, social, and structural factors driving these trends and to inform targeted prevention strategies [38].

In the symptom network linking depression, anxiety, and loneliness, the depressive symptom *‘anhedonia’* exhibited the strongest association with the anxiety symptom *‘feeling nervous’*, followed by *‘feeling tired’* and *‘irritable’*. Among the loneliness symptoms, *‘lacking companionship’* demonstrated a significant correlation with *‘feeling nervous’*. These findings are consistent with previous research in MSM, highlighting the frequent co-occurrence of depression, anxiety, and loneliness [17, 27]. Additionally, *‘feeling nervous’*, *‘feeling tired’* and *‘lacking companionship’* emerged as highly central nodes within the psychosocial symptom network in our study. Since centrality identifies symptoms that hold key positions and exert significant influence over the network, targeting these core symptoms in interventions may be an effective strategy to alleviate co-occurring mental health burdens among MSM.

In our network analysis, the desire for uncommitted sexual relationships was also identified as a central symptom within the network. Notably, among the associated factors, *‘lacking companionship’* showed the strongest connection with this desire, followed by *‘feeling nervous’*. This suggests that loneliness and anxiety were strongly linked to unrestricted sociosexual orientation, potentially serving as coping mechanisms to alleviate feelings of disconnection. Previous research has similarly indicated that MSM experiencing loneliness may seek physical intimacy as a substitute for emotional support [18]. In line with this, a study of 394 young adults found that individuals with poor mental health experienced temporary relief from depressive and loneliness symptoms following non-restrictive sexual behaviors [68]. Conversely, other studies have suggested that psychological issues such as depression, anxiety, and loneliness may result from casual sexual encounters. For example, a multi-ethnic study of 3,907 single, heterosexual college students across 30 U.S. universities reported that unrestricted sexual behavior was positively associated with psychological distress and negatively associated with well-being [69]. In addition, our network analysis revealed that the self-esteem items *‘satisfied with myself’* and *‘respect for myself’* were negatively associated with unrestricted sexual desire and attitudes, respectively. This pattern suggests that a higher level of sociosexual orientation may be linked to lower levels of self-esteem among MSM, consistent with findings in the general population [70]. However, the causal relationship between low self-esteem and non-restrictive sexual behavior remains unclear and warrants further longitudinal investigation. These findings need to be interpreted in the specific Chinese context. In China, strong cultural expectations of heterosexual marriage and family formation, together with persistent stigma toward same-sex behavior [71], may exacerbate feelings of loneliness and anxiety among MSM, thereby helping to explain the observed associations between unrestricted sexual desire and attitudes and psychosocial distress. Moreover, the limited availability of culturally sensitive mental health services and the need for many MSM to conceal their sexual identity may further undermine self-esteem. Rapid urbanization and the widespread use of online dating platforms have also reshaped partner-seeking patterns [72], facilitating casual sexual encounters but potentially increasing emotional vulnerability when such encounters fail to provide sustained social support.

Internalized homonegativity describes the self-loathing experienced by homosexual individuals. In this study, internalized homonegativity was assessed using three dimensions: social comfort with gay men, public identification as gay, and personal comfort with a gay identity. Network analysis revealed a negative association between social comfort with gay men and the desire for uncommitted sexual relationships. Consistent with prior research, Brian et al. [73] reported that higher levels of internalized homophobia were associated with stronger sexual urges, which in turn increased the likelihood of engaging in high-risk sexual behaviors. Beyond behavioral correlates, internalized homonegativity has been consistently linked to psychological problems. Newcomb et al. [74] found that internalized homophobia was associated with stronger suicidal ideation, more severe depressive symptoms, and reduced levels of help-seeking and perceived social support. Similarly, Bingham et al. [75] identified a significant negative association between internalized homophobia and self-esteem. Our network analysis further revealed a positive association between family support and personal comfort with a gay identity, suggesting that familial acceptance may play a protective role against internalized homonegativity. The attitudes and reactions of family members are closely tied to how individuals construct and accept their sexual identities. Negative parental responses to a child's sexual orientation have been shown to hinder self-acceptance and increase the risk of internalized stigma [76]. In the Chinese sociocultural context, where traditional norms surrounding marriage and sexuality remain strong, internalized homonegativity has been reported as a common challenge among MSM [35]. Such stigma may contribute to emotional distress and social isolation, which in some cases has been associated with greater involvement in high-risk sexual behaviors as a coping or validation strategy. These findings underscore the importance of family-inclusive interventions and culturally sensitive approaches that target both psychological and social barriers faced by MSM in China.

This study found that the psychosocial symptom networks varied across age groups among MSM. Specifically, compared to younger participants, older MSM exhibited stronger associations between depressive and anxiety symptoms, such as between *‘feeling tired’* and *‘irritable’*, *‘psychomotor symptoms’* and *‘cannot stop worrying’*, as well as *‘sleep problems’* and *‘feeling nervous’*. In contrast, the interconnections among loneliness, self-esteem, perceived social support, sociosexual orientation, and internalized homonegativity were notably weaker in the older group. This pattern may reflect cultural and structural factors, as older MSM in China often face stronger pressure to conceal their sexual identity due to marriage and family expectations [77]. This concealment may limit opportunities for peer connection and social support. Moreover, community-based interventions and online platforms that provide psychosocial resources are more often tailored toward younger MSM, leaving older MSM with fewer accessible support networks. These generational differences in cultural context and resource availability may help explain why psychosocial networks appeared less cohesive among older MSM. In the older MSM network, *‘feeling afraid’* and *‘satisfied with myself’* demonstrated higher betweenness centrality, suggesting that these anxiety and self-esteem symptoms may act as critical mediators of broader psychological distress in this subgroup. Conversely, in the younger group, *‘suicidal thoughts’*, loneliness-related items, and the desire for uncommitted sexual relationships showed higher centrality values than in older participants. These findings indicate that emotional isolation, impulsive sexual behavior, and suicidal ideation are more prominent features in the psychosocial profile of younger MSM, underscoring the need for age-sensitive mental health and behavioral interventions.

Importantly, regression analysis in this study demonstrated that central symptoms within the psychosocial network were significantly associated with high-risk sexual behaviors among MSM, with stronger associations observed in younger participants than in older one. These results suggest that psychological distress and sexual risk behaviors are interrelated, though the directionality of this relationship remains unclear. It is also plausible that engaging in high-risk sexual behaviors contributes to increased psychosocial burden, highlighting the need for longitudinal studies to disentangle these pathways. These results align with previous studies among MSM in China, which found that multiple psychosocial problems were associated with unprotected anal intercourse [34], and that MSM with five or more psychosocial problems had greater odds of having multiple sexual partners compared with those without syndemic conditions [27]. While these findings are interpreted within the Chinese sociocultural context, they also resonate with international research documenting strong links between loneliness [39], low self-esteem [78], and sexual risk-taking among MSM. At the same time, the influence of family expectations and stigma observed in this study may be more pronounced in China compared to many Western settings, underscoring the importance of cultural context in shaping psychosocial networks. Several mechanisms may explain the link between psychosocial problems and high-risk sexual behaviors. First, some individuals—particularly youth—may engage in sexual activity as a way to cope with psychological distress, escape unpleasant emotions or circumstances, or seek affirmation of their self-worth [79]. Second, negative emotional states, such as depression or anxiety, may impair judgment and decision-making, thereby increasing susceptibility to risky sexual encounters [80]. Even a negative affective state, in the absence of other clusters of depressive symptomatology, can still result in a less severe, but impaired decision making [81]. Third, MSM experiencing serious psychological distress may have a history of complex trauma in their childhood or adolescent development, which is often associated with a higher likelihood of engaging in unsafe sexual practices [82]. Such behaviors may serve as maladaptive coping mechanisms the individual has developed over time. Given these findings, targeting central symptoms within the psychosocial network of MSM may serve as an effective intervention strategy to reduce high-risk sexual behaviors and, consequently, lower the risk of HIV and other sexually transmitted infections in this vulnerable population. Integrative interventions that simultaneously address psychological and social factors are essential to effectively mitigate these risks.

### Strengths and limitations

This study has several strengths. By applying network analysis to simultaneously examine seven psychosocial problems within a Chinese MSM population, it contributes to a more comprehensive understanding of the psychosocial mechanisms that may be associated with high-risk sexual behaviors. The use of age-stratified network analyses further highlights the heterogeneity of psychosocial patterns across subgroups, offering valuable insights for the design of age-tailored interventions. Additionally, the identification of central symptoms linked to high-risk sexual behaviors provides a potential theoretical basis for developing targeted mental health and behavioral interventions.

However, several limitations should be acknowledged. First, the cross-sectional design precludes causal inferences regarding the relationships between psychosocial symptoms and high-risk sexual behaviors. Second, participants were recruited through venue-based and online snowball sampling, which may lead to a bias toward more socially connected MSM. Furthermore, as the study was conducted in Taizhou, the findings may not be fully generalizable to the broader MSM population in China or other regions with different socio-cultural contexts. Further research with diverse, nationally representative samples is needed to validate these results and assess their broader applicability. Third, self-reported data on psychosocial problems and sexual behaviors are subject to recall bias and social desirability bias, which may have led to underreporting of sensitive behaviors. Finally, although network analysis highlights central symptoms and interconnections, it does not capture the temporal dynamics of symptom interactions, which warrant further investigation using longitudinal or ecological momentary assessment designs. Future studies should adopt multi-site, longitudinal, and mixed-method approaches to validate and extend these findings.

## Conclusions

In conclusion, this study mapped the network structure and interconnections among key psychosocial factors—including depression, anxiety, self-esteem, internalized homonegativity, loneliness, perceived social support, and sociosexual orientation—across different age groups of MSM. Central symptoms such as *‘feeling nervous’*, desire for uncommitted sexual relationships, *‘feeling tired’*, *‘lacking companionship’,* and *‘no good’* played particularly influential roles within the psychosocial network and were significantly associated with high-risk sexual behaviors. Notably, the relationship between psychosocial problems and sexual risk-taking appeared more pronounced among younger MSM. These findings offer a theoretical foundation for the development of future interventions aimed at improving mental health and reducing high-risk sexual behaviors within the MSM population.

## Supplementary Information

Supplementary Material 1.

## Data Availability

Data and materials are available on request from the corresponding author.

## Abbreviations

MSM: Men who have sex with men
UAI: Unprotected anal intercourse
MSP: Multiple sexual partners
HIV: Human immunodeficiency virus
STIs: Sexually transmitted infections
CI: Confidence interval
PHQ-9: Patient health questionnaire-9 scale
GAD-7: Generalized anxiety disorder 7-item scale
RSES: Rosenberg self-esteem scale
SIHS: Short internalized homonegativity scale
UCLA-3: 3-Item UCLA loneliness scale
MSPSS: Multidimensional scale of perceived social support
SOI-R: The revised sociosexual orientation inventory
EI: Expected influence
LOWESS: Locally weighted scatterplot smoothing
SIHSSC: Subscale of short internalized homonegativity scale: "Social Comfort with Gay Men"
SIHSPUBID: Subscale of short internalized homonegativity scale: "Public Identification as Gay"
SIHSPC: Subscale of short internalized homonegativity scale: "Personal Comfort with a Gay Identity
