# Correction to: Psychosocial symptom networks and high-risk sexual behaviors among men who have sex with men: a network analysis

**DOI:** 10.1186/s40359-025-03753-2

**Published:** 2025-11-27

**Authors:** Nan Lin, Yuan Guo, Yun Chen, Yuting Yang, Haijiang Lin, Xiaoxiao Chen, Tingting Wang, Chaowei Fu, Shanling Wang, Jingyi Wang

**Affiliations:** 1 School of Public Health, NHC Key Laboratory of Health Technology Assessment, Fudan University, Shanghai, China; 2 Yale School of Nursing, Orange, CT 06477 USA; 3 Taizhou City Center for Disease Control and Prevention, Taizhou, Zhejiang Province China; 4 Taizhou Central Blood Station, Taizhou, Zhejiang Province China

**Lin et al. BMC Psychology 13, 1252 (2025)**

**https://doi.org/10.1186/s40359-025-03550-x**

Following the publication of the original article, the authors reported that Shanling Wang and Jingyi Wang should have been designated as co-corresponding authors. This indication was missing from the original article and has now been included in this correction.

The original article has been updated accordingly.

