# Supplementary material for: Psychosocial symptom networks and high-risk sexual behaviors among men who have sex with men: a network analysis
Source: BMC Psychol. 2025 Nov 12;13:1252. doi: 10.1186/s40359-025-03550-x (PMC12613908; doi:10.1186/s40359-025-03550-x)
Supplement: Supplementary file 1 — Supplementary Material 1. [file 40359_2025_3550_MOESM1_ESM.pdf]

Psychosocial symptom networks and high-risk sexual behaviors among men who have sex with men: a network analysis

Additional file

Table S1 Questionnaires and items used for the assessment of psychosocial problems

	Reference Name	Item
**PHQ-9**	Anhedonia	Little interest or pleasure in doing things
	Sad Mood	Feeling down, depressed, or hopeless
	Sleep problems	Trouble falling or staying asleep, or sleeping too much
	Feeling tired	Feeling tired or having little energy
	Appetite problems	Poor appetite or overeating
	Feeling guilty	Feeling bad about yourself - or that you are a failure or have let yourself or your family down
	Trouble concentrating	Trouble concentrating on things, such as reading the newspaper or watching television
	Psychomotor symptoms	Moving or speaking so slowly that other people could have noticed. Or the opposite - being so fidgety or restless that you have been moving around a lot more than usual
	Suicidal thoughts	Thoughts that you would be better off dead, or of hurting yourself in some way
**GAD-7**	Feeling nervous	Feeling nervous, anxious, or on edge
	Cannot stop worrying	Not being able to stop or control worrying
	Worrying too much	Worrying too much about different things
	Trouble relaxing	Trouble relaxing
	Restless	Being so restless that it is hard to sit still
	Irritable	Becoming easily annoyed or irritable
	Feeling afraid	Feeling afraid as if something awful might happen
**UCLA-3**	Lacking companionship	How often do you feel that you lack companionship?
	Feeling left out	How often do you feel left out?
	Feeling isolated	How often do you feel isolated from others?

Table S1 Questionnaires and items used for the assessment of psychosocial problems (continued)

	Reference Name	Item
**RSES**	Worth	I feel that I am a person of worth, at least on an equal plane with others
	Good qualities	I feel that I have a number of good qualities
	Failure	All in all, I am inclined to feel that I am a failure*
	Doing things well	I am able to do things as well as most people
	Proud	I feel I do not have much to be proud of*
	Positive attitude	I take a positive attitude toward myself
	Satisfied with myself	On the whole, I am satisfied with myself
	Respect for myself	I wish I could have more respect for myself*
	Feeling useless	I certainly feel useless at times*
	No good	At times I think that I am no good at all*
**MSPSS**	MSPSS family subscale	My family really tries to help me
		I get the emotional help & support I need from my family
		I can talk about my problems with my family
		My family is willing to help me make decisions
	MSPSS friends subscale	My friends really try to help me
		I can count on my friends when things go wrong
		I have friends with whom I can share my joys and sorrows
		I can talk about my problems with my friends
	MSPSS significant other subscale	There is a special person who is around when I am in need
		There is a special person with whom I can share joys and sorrows
		I have a special person who is a real source of comfort to me
		There is a special person in my life who cares about my feelings

Table S1 Questionnaires and items used for the assessment of psychosocial problems (continued)

	Reference Name	Item
**SOI-R**	SOI-R attitude facet	Sex without love is OK
		I can imagine myself being comfortable and enjoying "casual" sex with different partners
		I do not want to have sex with a person until I am sure that we will have a long-term, serious relationship*
	SOI-R desire facet	How often do you have fantasies about having sex with someone you are not in a committed romantic relationship with?
		How often do you experience sexual arousal when you are in contact with someone you are not in a committed romantic relationship with?
		In everyday life, how often do you have spontaneous fantasies about having sex with someone you have just met?
**SIHS**	SIHS social comfort with gay men (SC) factor	Obviously effeminate homosexual men make me feel uncomfortable*
		I feel comfortable in gay bars
		Social situations with gay men make me feel uncomfortable*
	SIHS public identification as gay (PUBID) factor	I feel comfortable being seen in public with an obviously gay person
		I feel comfortable discussing homosexuality in a public situation
	SIHS personal comfort with a gay identity (PC) factor	I feel comfortable being a homosexual man
		Homosexuality is morally acceptable to me
		Even if I could change my sexual orientation, I wouldn't

PHQ-9: Patient Health Questionnaire-9; GAD-7: Generalized Anxiety Disorder-7; UCLA-3: UCLA 3-Item Loneliness Scale; RSES: Rosenberg Self-Esteem Scale; MSPSS: Multidimensional Scale of Perceived Social Support; SOI-R: Revised Sociosexual Orientation Inventory; SIHS: Short Internalized Homonegativity Scale

* Reversed-coded item

Table S2 Estimated edge weights of the network

	Anhedonia	Sad Mood	Sleep problems	Feeling tired	Appetite problems	Feeling guilty	Trouble concentrating	Psychomotor symptoms	Suicidal thoughts	Feeling nervous	Cannot stop worrying	Worrying too much	Trouble relaxing	Restless	Irritable	Feeling afraid	Lacking companionship	Feeling left out
Anhedonia	NA	0.26	0.08	0.12	NA	NA	0.12	NA	NA	0.23	NA	0.09	NA	NA	NA	NA	NA	NA
Sad Mood	0.26	NA	0.12	0.08	NA	0.10	NA	0.09	0.07	NA	NA	NA	NA	0.05	0.05	NA	NA	0.05
Sleep problems	0.08	0.12	NA	0.21	NA	NA	NA	NA	NA	NA	0.11	NA	NA	NA	NA	0.05	NA	NA
Feeling tired	0.12	0.08	0.21	NA	0.13	NA	0.07	NA	NA	NA	0.05	0.06	NA	NA	0.16	NA	NA	NA
Appetite problems	NA	NA	NA	0.13	NA	0.15	0.10	0.11	NA	0.05	NA	NA	NA	NA	NA	NA	NA	NA
Feeling guilty	NA	0.10	NA	NA	0.15	NA	0.12	0.09	0.10	0.08	NA	NA	0.13	0.09	NA	NA	NA	NA
Trouble concentrating	0.12	NA	NA	0.07	0.10	0.12	NA	0.16	0.02	NA	0.05	NA	0.05	0.08	NA	0.07	NA	NA
Psychomotor symptoms	NA	0.09	NA	NA	0.11	0.09	0.16	NA	0.16	NA	0.07	NA	NA	0.10	NA	NA	NA	NA
Suicidal thoughts	NA	0.07	NA	NA	NA	0.10	NA	0.16	NA	NA	0.06	NA	NA	NA	NA	0.13	NA	NA
Feeling nervous	0.23	NA	NA	NA	0.05	0.08	NA	NA	NA	NA	0.26	0.13	NA	0.07	0.09	NA	0.09	NA
Cannot stop worrying	NA	NA	0.11	0.05	NA	NA	0.05	0.07	0.06	0.26	NA	0.10	NA	0.15	0.12	NA	NA	NA
Worrying too much	0.09	NA	NA	0.06	NA	NA	NA	NA	NA	0.13	0.10	NA	0.20	NA	0.12	0.05	NA	NA
Trouble relaxing	NA	NA	NA	NA	NA	0.13	0.05	NA	NA	NA	NA	0.20	NA	0.16	0.08	0.12	NA	NA
Restless	NA	0.05	NA	NA	NA	0.09	0.08	0.10	NA	0.07	0.15	NA	0.16	NA	NA	0.19	NA	NA
Irritable	NA	0.05	NA	0.16	NA	NA	NA	NA	NA	0.09	0.12	0.12	0.08	NA	NA	0.12	NA	NA
Feeling afraid	NA	NA	0.05	NA	NA	NA	0.07	NA	0.13	NA	NA	0.05	0.12	0.19	0.12	NA	NA	NA
Lacking companionship	NA	NA	NA	NA	NA	NA	NA	NA	NA	0.09	NA	NA	NA	NA	NA	NA	NA	0.26
Feeling left out	NA	0.05	NA	NA	NA	NA	NA	NA	NA	NA	NA	NA	NA	NA	NA	NA	0.26	NA
Feeling isolated	NA	NA	NA	NA	NA	NA	NA	NA	NA	NA	NA	NA	NA	NA	NA	NA	0.27	0.45
Worth	NA	NA	NA	NA	NA	NA	NA	NA	NA	NA	NA	NA	NA	NA	NA	NA	NA	NA

Table S2 Estimated edge weights of the network (continued)

	Anhedonia	Sad Mood	Sleep problems	Feeling tired	Appetite problems	Feeling guilty	Trouble concentrating	Psychomotor symptoms	Suicidal thoughts	Feeling nervous	Cannot stop worrying	Worrying too much	Trouble relaxing	Restless	Irritable	Feeling afraid	Lacking companionship	Feeling left out
Good qualities	NA	NA	NA	NA	NA	NA	NA	NA	NA	NA	NA	NA	NA	NA	NA	NA	NA	NA
Failure	NA	NA	NA	NA	NA	NA	NA	NA	NA	NA	NA	NA	NA	NA	NA	-0.05	NA	NA
Doing things well	NA	NA	NA	NA	NA	NA	NA	NA	NA	NA	NA	NA	NA	NA	NA	NA	NA	NA
Proud	NA	NA	NA	NA	NA	NA	NA	NA	NA	NA	NA	NA	NA	NA	NA	NA	NA	NA
Positive attitude	NA	NA	NA	NA	NA	NA	NA	NA	NA	NA	NA	NA	NA	NA	NA	NA	NA	NA
Satisfied with myself	NA	NA	NA	NA	NA	NA	NA	NA	NA	NA	NA	NA	NA	NA	NA	NA	NA	NA
Respect for myself	NA	-0.05	NA	NA	NA	NA	NA	NA	NA	NA	NA	-0.06	NA	-0.05	NA	NA	NA	NA
Feeling useless	NA	NA	NA	NA	NA	NA	NA	NA	NA	NA	NA	NA	NA	NA	NA	NA	NA	NA
No good	NA	NA	NA	NA	NA	NA	NA	NA	NA	NA	NA	NA	NA	NA	NA	NA	NA	NA
MSPSS family subscale	NA	NA	NA	NA	NA	NA	NA	NA	NA	NA	NA	NA	NA	NA	NA	NA	NA	NA
MSPSS friends subscale	NA	NA	NA	NA	NA	NA	NA	NA	NA	NA	NA	NA	NA	NA	NA	NA	NA	NA
MSPSS significant other subscale	NA	NA	NA	NA	NA	NA	NA	NA	NA	NA	NA	NA	NA	NA	NA	NA	NA	-0.07
SOI-R attitude facet	0.06	NA	NA	NA	NA	NA	NA	NA	-0.05	0.07	NA	NA	NA	NA	NA	NA	NA	NA
SOI-R desire facet	NA	NA	NA	-0.09	NA	NA	NA	NA	NA	0.06	NA	NA	NA	NA	NA	NA	0.25	NA
SIHS social comfort with gay men (SC) factor	NA	NA	NA	NA	NA	-0.07	NA	NA	NA	NA	NA	NA	0.07	NA	NA	NA	NA	NA
SIHS public identification as gay (PUBID) factor	NA	NA	NA	NA	NA	NA	NA	NA	NA	NA	0.01	NA	0.05	NA	0.07	NA	NA	NA
SIHS personal comfort with a gay identity (PC) factor	NA	NA	NA	0.09	NA	NA	NA	NA	NA	NA	NA	NA	NA	NA	NA	NA	NA	NA

Table S2 Estimated edge weights of the network (continued)

	Feeling isolated	Good qualities	Failure	Doing things well	Proud	Positive attitude	Satisfied with myself	Respect for myself	Feeling useless	No good	MSPSS family subscale	MSPSS friends subscale	MSPSS significant other subscale	SOI-R attitude facet	SOI-R desire facet	SIHS social comfort with gay men (SC) factor	SIHS public identification as gay (PUBID) factor	SIHS personal comfort with a gay identity (PC) factor
Anhedonia	NA	NA	NA	NA	NA	NA	NA	NA	NA	NA	NA	NA	NA	0.06	NA	NA	NA	NA
Sad Mood	NA	NA	NA	NA	NA	NA	NA	-0.05	NA	NA	NA	NA	NA	NA	NA	NA	NA	NA
Sleep problems	NA	NA	NA	NA	NA	NA	NA	NA	NA	NA	NA	NA	NA	NA	NA	NA	NA	NA
Feeling tired	NA	NA	NA	NA	NA	NA	NA	NA	NA	NA	NA	NA	NA	NA	-0.09	NA	NA	0.09
Appetite problems	NA	NA	NA	NA	NA	NA	NA	NA	NA	NA	NA	NA	NA	NA	NA	NA	NA	NA
Feeling guilty	NA	NA	NA	NA	NA	NA	NA	NA	NA	NA	NA	NA	NA	NA	NA	-0.07	NA	NA
Trouble concentrating	NA	NA	NA	NA	NA	NA	NA	NA	NA	NA	NA	NA	NA	NA	NA	NA	NA	NA
Psychomotor symptoms	NA	NA	NA	NA	NA	NA	NA	NA	NA	NA	NA	NA	NA	NA	NA	NA	NA	NA
Suicidal thoughts	NA	NA	NA	NA	NA	NA	NA	NA	NA	NA	NA	NA	NA	-0.05	NA	NA	NA	NA
Feeling nervous	NA	NA	NA	NA	NA	NA	NA	NA	NA	NA	NA	NA	NA	0.07	0.06	NA	NA	NA
Cannot stop worrying	NA	NA	NA	NA	NA	NA	NA	NA	NA	NA	NA	NA	NA	NA	NA	NA	NA	NA
Worrying too much	NA	NA	NA	NA	NA	NA	NA	-0.06	NA	NA	NA	NA	NA	NA	NA	NA	NA	NA
Trouble relaxing	NA	NA	NA	NA	NA	NA	NA	NA	NA	NA	NA	NA	NA	NA	NA	0.07	0.05	NA
Restless	NA	NA	NA	NA	NA	NA	NA	-0.05	NA	NA	NA	NA	NA	NA	NA	NA	NA	NA
Irritable	NA	NA	NA	NA	NA	NA	NA	NA	NA	NA	NA	NA	NA	NA	NA	NA	0.07	NA
Feeling afraid	NA	NA	NA	-0.05	NA	NA	NA	NA	NA	NA	NA	NA	NA	NA	NA	NA	NA	NA
Lacking companionship	0.27	NA	NA	NA	NA	NA	NA	NA	NA	NA	NA	NA	NA	NA	0.25	NA	NA	NA
Feeling left out	0.45	NA	NA	NA	NA	NA	NA	NA	NA	NA	NA	NA	-0.07	NA	NA	NA	NA	NA
Feeling isolated	NA	NA	NA	NA	NA	NA	-0.06	NA	NA	NA	NA	NA	NA	NA	NA	NA	NA	NA
Worth	NA	NA	0.51	NA	0.06	0.10	NA	NA	NA	NA	0.05	NA	NA	NA	NA	NA	NA	NA

Table S2 Estimated edge weights of the network (continued)

	Feeling isolated	Good qualities	Failure	Doing things well	Proud	Positive attitude	Satisfied with myself	Respect for myself	Feeling useless	No good	MSPSS family subscale	MSPSS friends subscale	MSPSS significant other subscale	SOI-R attitude facet	SOI-R desire facet	SIHS social comfort with gay men (SC) factor	identification as gay (PUBID) factor	comfort with a gay identity (PC) factor
Good qualities	NA	0.51	NA	NA	NA	0.08	0.11	NA	NA	0.05	NA	0.05	NA	NA	NA	NA	NA	NA
Failure	NA	NA	NA	NA	0.25	NA	0.06	NA	0.05	0.29	NA	NA	NA	NA	NA	NA	NA	NA
Doing things well	NA	0.09	0.29	NA	0.05	0.28	0.05	NA	NA	NA	NA	NA	NA	NA	NA	NA	NA	NA
Proud	NA	0.06	NA	0.25	NA	NA	NA	0.11	0.05	0.08	NA	0.06	NA	NA	NA	NA	NA	NA
Positive attitude	NA	0.10	0.08	NA	NA	NA	0.35	NA	0.06	NA	NA	NA	0.05	NA	NA	NA	NA	NA
Satisfied with myself	-0.06	NA	0.11	0.06	NA	0.35	NA	NA	NA	NA	NA	0.05	0.07	NA	-0.10	NA	NA	NA
Respect for myself	NA	NA	NA	NA	0.11	NA	NA	NA	0.15	NA	NA	NA	NA	-0.10	NA	-0.11	NA	NA
Feeling useless	NA	NA	NA	0.05	0.05	0.06	NA	0.15	NA	0.66	NA	NA	NA	NA	NA	NA	NA	NA
No good	NA	NA	0.05	0.29	0.08	NA	NA	NA	0.66	NA	NA	NA	NA	NA	0.07	NA	NA	NA
MSPSS family subscale	NA	0.05	NA	NA	NA	NA	NA	NA	NA	NA	NA	0.36	0.41	NA	NA	NA	0.05	0.08
MSPSS friends subscale	NA	NA	0.05	NA	0.06	NA	0.05	NA	NA	NA	0.36	NA	0.36	-0.06	NA	NA	NA	NA
MSPSS significant other subscale	NA	NA	NA	NA	NA	0.05	0.07	NA	NA	NA	0.41	0.36	NA	NA	-0.07	NA	NA	NA
SOI-R attitude facet	NA	NA	NA	NA	NA	NA	NA	-0.10	NA	NA	NA	-0.06	NA	NA	0.41	NA	0.11	NA
SOI-R desire facet	NA	NA	NA	NA	NA	NA	-0.10	NA	NA	0.07	NA	NA	-0.07	0.41	NA	-0.12	NA	NA
SIHS social comfort with gay men (SC) factor	NA	NA	NA	NA	NA	NA	NA	-0.11	NA	NA	NA	NA	NA	NA	-0.12	NA	NA	0.08
SIHS public identification as gay (PUBID) factor	NA	NA	NA	NA	NA	NA	NA	NA	NA	NA	0.05	NA	NA	0.11	NA	NA	NA	0.46
SIHS personal comfort with a gay identity (PC) factor	NA	NA	NA	NA	NA	NA	NA	NA	NA	NA	0.08	NA	NA	NA	NA	0.08	0.46	NA

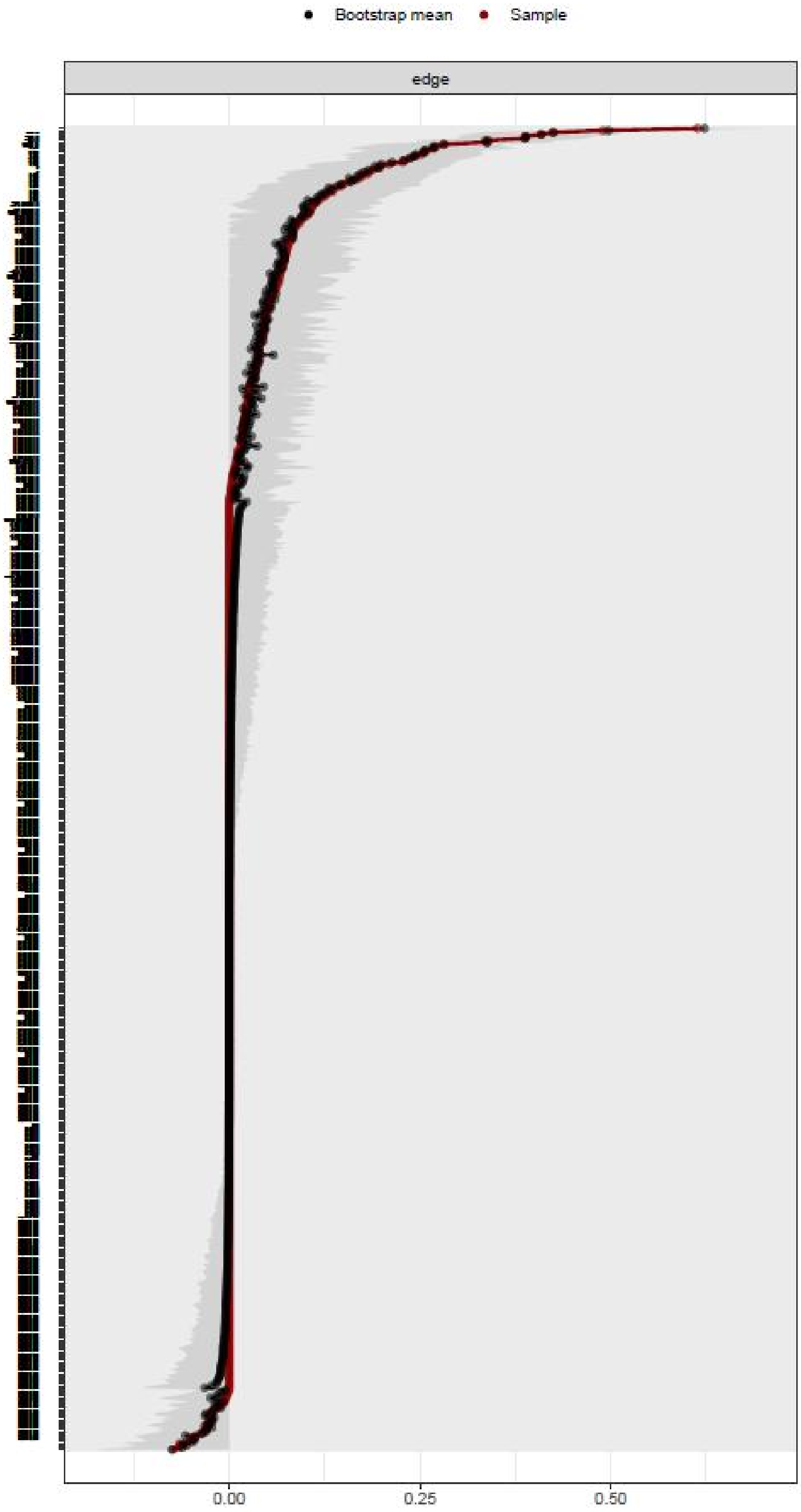

Figure S1 Stability of edge weights

The x-axle indicates the edge weights and the y-axle indicates the nodes linked by the edges. The black dots denote the mean value of the bootstrapped edge weights and the red dots denote the edge weights from current sample. The black lines denote the 95% confidence intervals of the bootstrapped sample.
